# Performance of Paracheck™-Pf, SD Bioline malaria Ag-Pf and SD Bioline malaria Ag-Pf/pan for diagnosis of falciparum malaria in the Central African Republic

**DOI:** 10.1186/1471-2334-14-109

**Published:** 2014-02-26

**Authors:** Djibrine Djallé, Jean Chrysostome Gody, Jean Methode Moyen, Gaspard Tekpa, Julienne Ipero, Nestor Madji, Sébastien Breurec, Alexandre Manirakiza

**Affiliations:** 1 Institut Pasteur de Bangui, PO Box 923, Bangui, Central African Republic; 2 Complexe Pédiatrique de Bangui, Ministry of Public Health, Population and AIDS Control, PO Box 883, Bangui, Central African Republic; 3 Malaria Programme Division, Ministry of Public Health, Population and AIDS Control, PO Box 883, Bangui, Central African Republic; 4 Hôpital de l’Amitié, Ministry of Public Health, Population and AIDS Control, PO Box 883, Bangui, Central African Republic

**Keywords:** Rapid diagnostic test, Malaria, Central African Republic

## Abstract

**Background:**

Rapid diagnostic tests (RDTs) are the current complement to microscopy for ensuring prompt malaria treatment. We determined the performance of three candidate RDTs (Paracheck™-Pf, SD Bioline malaria Ag-Pf and SD Bioline malaria Ag-Pf/pan) for rapid diagnosis of malaria in the Central African Republic.

**Methods:**

Blood samples from consecutive febrile patients who attended for laboratory analysis of malaria at the three main health centres of Bangui were screened by microscopy and the RDTs. Two reference standards were used to assess the performance of the RDTs: microscopy and, a combination of microscopy plus nested PCR for slides reported as negative, on the assumption that negative results by microscopy were due to sub-patent parasitaemia.

**Results:**

We analysed 436 samples. Using the combined reference standard of microscopy + PCR, the sensitivity of Paracheck™-Pf was 85.7% (95% CI, 80.8–89.8%), that of SD Bioline Ag-Pf was 85.4% (95% CI, 80.5–90.7%), and that of SD Bioline Ag-Pf/pan was 88.2% (95% CI, 83.2–92.0%). The tests performed less well in cases of low parasitaemia; however, the sensitivity was > 95% at > 500 parasites/μl.

**Conclusions:**

Overall, SD Bioline malaria Ag-Pf and SD Bioline malaria Ag-Pf/pan performed slightly better than Paracheck™-Pf. Use of RDTs with reinforced microscopy practice and laboratory quality assurance should improve malaria treatment in the Central African Republic.

## Background

In countries in sub-Saharan Africa, malaria management is frequently based on clinical criteria, leading to overuse of antimalarial agents [1,2]. After the 1990s, a rapid increase in resistance to first-line therapy was observed, with a consequent decrease in the efficacy of chloroquine [3] and its replacement by sulfadoxine-pyrimethamine [4,5]. Subsequently, effective but more expensive artemisinin combination treatments were introduced for first-line treatment [6]. These new therapies should be targeted in order to avoid overuse of antimalarial drugs, which can lead to the selection of drug-resistant parasites [7].

The World Health Organization (WHO) recommends parasitological confirmation for all patients suspected of having malaria before starting treatment. This recommendation includes high-quality microscopy or, when that is not available, use of easy-to-use rapid diagnostic tests (RDTs) [8]. Panel studies have been conducted to compare the performance of commercially available RDTs [9]. Although these studies provide selection criteria, field evaluations are essential to obtain operational data [10-15], as the heterogeneous performance of these tests depends on local factors, such as deletions or mutations in the *PfHRP-2* gene [16-21], the prozone effect [22], cross-reactivity with human autoantibodies [23,24] and the presence of other infectious diseases [25]. Local data are therefore required to select suitable RDTs before they are used [16,26-28].

A recent multi-centre study assessing global sequence variation in *PfHRP-2* and −*3* found wide variation in *PfHRP-2* sequences in samples from the Central African Republic. The authors speculated that this might reduce the sensitivity of RDTs for detecting malaria in people with very low parasite density [29]. In this country, the quality of microscopy in the public health sector is poor, and presumptive treatment of malaria is still widespread [30]. The Ministry of Health has selected the RDTs Paracheck™-Pf, SD Bioline malaria Ag-Pf and SD Bioline malaria Ag-Pf/pan as candidates on the basis of panel studies [9]. The objective of the study reported here was to evaluate the performance of these tests in the field.

## Methods

### Study area and design

This cross-sectional study was conducted in August 2011 in Bangui, capital of the Central African Republic, in the Hospital de l’Amitié, the Complexe Pediatrique and the Institut Pasteur of Bangui. Bangui is located beside the Oubangui River, north of the Democratic Republic of the Congo (geographical coordinates 7.00 N, 21.00 E). The climate is tropical, and rainfall peaks between April and November. The average temperature varies from 19°C to 32°C. The main malaria parasite is *Plasmodium falciparum*, and malaria transmission occurs throughout the year, with peaks at the beginning and the end of the rainy season, although no data are available on the intensity of transmission. Malaria accounts for more than 40% of morbidity in the country (Central African Republic Ministry of Health, 2010 annual report, unpublished data).

The Hospital de l’Amitié and the Complexe Pédiatrique are tertiary referral public health centres equipped with laboratories where thin and thick smears are analysed. The Institut Pasteur of Bangui is a private centre (International Network of Instituts Pasteur) for biomedical research, in which laboratory diagnosis for malaria is performed for patients referred by clinicians at health centres where this test is not available.

We analysed the performance of three RDTs: Paracheck™-Pf, SD Bioline malaria Ag-Pf and SD Bioline malaria Ag-Pf and /pan (Standard Diagnostics Ref 05FK60, Inc; Suwon City, Republic of Korea). SD Bioline Malaria Ag Pf and Paracheck™-Pf contain antibodies against *P. falciparum*-specific histidine-rich protein type 2 (PfHRP2), while Bioline Malaria Ag Pf/Pan contains antibodies targeting both PfHRP2 and lactate dehydrogenase specific to *P. falciparum* and other *Plasmodium* species (*P. vivax*, *P. ovale* and *P. malariae*). Blood samples from people attending each study centre for laboratory analysis were tested for the presence or absence of malaria parasites by microscopy and the RDTs. Samples from patients with negative microscopy (regardless of RDT result) were tested *P. falciparum* by nested PCR, on the assumption that false-positive results with RDTs are due to sub-patent parasitaemia [31].

### Patient recruitment

Consecutive patients of all ages presenting at each study site were approached by the study technician for recruitment. Each patient’s medical card was checked, and those with a request for smear analysis were considered eligible if they were febrile (axillary temperature ≥ 37.5°C) or had a history of fever in the previous 24 h. They were included in the study if they gave written consent; for patients aged ≤ 18 years, informed consent was provided by a parent or guardian.

### Ethical approval

This project was reviewed and approved by the ethical committee of the University of Bangui. The Central African Republic Health Ministry also gave approval for this study.

### Sample size estimation

For an estimated annual malaria rate in Bangui of 40% (Central African Republic Ministry of Health, 2010 annual report, unpublished data), an expected sensitivity of Bioline RDTs of 95% [26] and a sensitivity set at ± 2.5%, a sample size of 421 patients was estimated.

### Selection of technicians

In each study centre, the laboratory team consisted of three technicians with university training, all of whom had been re-trained for diagnosing malaria in the laboratory by standard operational procedures [32]. The technicians were also trained to perform and read the Paracheck™-Pf, SD Bioline malaria Ag-Pf and SD Bioline malaria Ag-Pf and /pan tests. At the Institut Pasteur, a fourth laboratory technician was designated to analyse all discrepant slides and to perform PCR.

### Malaria diagnosis

Finger-prick blood samples were obtained for slides and for testing with the RDTs. Blood smears were air-dried, stained with 4% Giemsa and analysed under a light microscope (× 100 oil immersion) to detect asexual forms of *P. falciparum*. Parasite density was determined as the number of parasites per 200 leukocytes on the assumption of an average leukocyte count of 8000/μl of blood. A result was considered negative if no parasites were detected per 200 leukocytes. Each slide was read independently by two study technicians. In the case of a discrepant qualitative result (negative or positive), a third reading was done by the designated technician at the Institut Pasteur. All the laboratory technicians were blinded to the RDT results. The results of both microscopy and the RDTs were reported to the clinicians, who were advised to treat the patient for malaria if the results of these two analyses were discrepant.

The RDTs were performed by the third technician at the study centre following the manufacturer’s instructions.

For patients with negative microscopy, three drops of blood were collected on a piece of filter paper (Whatman®). The blood spots were air-dried and stored at 4°C in individual sterile plastic bags for PCR analysis.

Parasite DNA extraction and PCR assays were performed at the Institut Pasteur. The blood-impregnated filter paper piece was washed with distilled water and placed directly in a PCR tube containing the PCR reaction components. Genomic DNA was determined in an assay based on nested PCR for *Plasmodium* DNA [33].

### Statistical analysis

Data were entered onto Excel spreadsheets and analysed with MedCalc®software (MedCalc Software, Acacialaan 22, B-8400 Ostend, Belgium).

The first step of our analysis was to determine the performance of the RDTs according to the falciparum density in samples found positive by microscopy. Density was categorized as ≤ 100, 101–200, 201–500, 501–1000, 1001–5000, 5001–50 000 and > 50 000 parasites/μl. RDT results were compared (paired proportions) with the McNemar test, at a level of significance (*P*) of 0.05.

The second step was to estimate the performance of the RDTs against the combined results of microscopy and nested PCR, expressed as true-positive (TP), true-negative (TN), false-positive (FP) or false negative (FN). The formulas used to calculate performance were TP/TP + FN for sensitivity, TN/TN + FP for specificity, TP/TP + FP for positive predictive value (PPV) and TN/TN + FN for negative predictive value (NPV). The results were interpreted with 95% confidence intervals (CIs). At baseline, the results of the comparison between the RDTs and microscopy were expressed as TP_1_, TN_1_, FP_1_ or FN_1_. Then, the analysis took onto account the results of PCR performed on samples that were negative by microscopy (classified as FP_1_ or TN_1_ in the RDTs). According to the PCR results, the results of the RDTs were designated as TP_2_ if PCR was positive in FP_1_, FP_2_ if PCR was negative in FP_1_, FN_2_ if PCR was positive in TN_1_ and TN_2_ if PCR was negative in TN_1_. Combination of the results of the RDTs for samples positive by microscopy and of PCR for samples negative by microscopy gave the values TP = TP_1_ + TP_2_, TN = TN_2_, FP = FP_2_ and FN = FN_1_ + FN_2_, which were used to calculate the performance of the RDTs.

## Results

A total of 437 patients were recruited for this study between 8 and 22 August 2011. Microscopic analysis showed that 53.8% (235/437) of the blood slides were positive for *P. falciparum*. In 234/235 (99.6%) cases of infection, *P. falciparum* was the only parasite species identified. One infected individual had a *P. ovale* mono-infection (detected by SD Bioline malaria Ag-Pf/pan and confirmed by nested PCR) and was hence excluded from the study, which was confined to detection of falciparum malaria.

The RDTs gave positive results in 52.3% (228/436) of cases with Paracheck™-Pf, 57.8% (252/436) with SD Bioline malaria Ag-Pf and 58.0% (253/436) with SD Bioline malaria Ag-Pf/pan.

Comparison of the results of the RDTs with those of microscopy showed concordant positive results in 85.9% (201/234) of cases with Paracheck™-Pf, 92.3% (216/234) with SD Bioline malaria Ag-Pf and 92.3% (216/234) with SD Bioline malaria Ag-Pf/pan. Of the samples that were negative by microscopy, 13.4% (27/202) were found to be positive with Paracheck™-Pf, 17.8% (36/202) with SD Bioline malaria Ag-Pf and 18.8% (38/202) with SD Bioline malaria Ag-Pf/pan.

**Figure 1:**
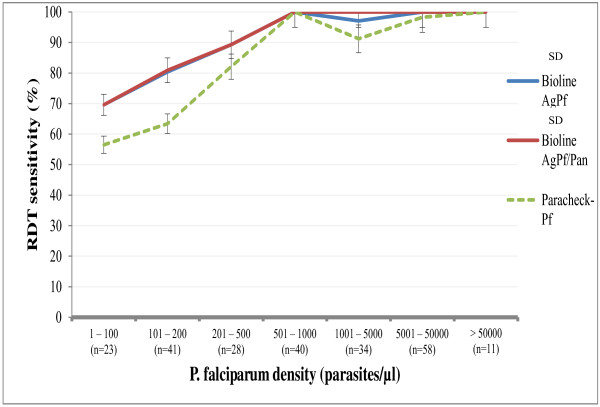
Rapid diagnostic test performance results by parasitaemia level.

The proportion of positive results with each of the RDTs increased with parasitaemia. For a parasitaemia ≤ 100 parasites/μl, the proportions were 56.5% with Paracheck™-Pf and 69.6% with SD Bioline malaria Ag-Pf and SD Bioline malaria Ag-Pf/pan. For a parasitaemia < 501 parasites/μl, the proportion of positive results with Paracheck™-Pf was significantly lower than with the Bioline kits (*p* = 0.04) (Figure 1). The sensitivity of the RDTs correlated positively with parasitaemia, with values < 70% when the parasitaemia was < 100 parasites/μl. The sensitivity of the SD Bioline devices increased to > 80% when the parasitaemia was 101–200 parasites/μl, whereas the sensitivity of Paracheck™-Pf remained at 63.4%. The sensitivity of all three tests increased substantially (> 95%) at parasite counts > 500 parasites/μl (Table 1). Negative results were found with all three RDTs in 18 blood samples with parasitaemia ranging from 40 to 400 parasites/μl.

**Table 1:** Sensitivity of rapid diagnostic tests by parasite density (microscopy)

**Parasites/μl (microscopy)**	**No. of analyses**	**SD Bioline AgPf**		**SD Bioline AgPf/Pan**		**Paracheck™-Pf**	
		**Sensitivity (%)**	**95% CI**	**Sensitivity (%)**	**95% CI**	**Sensitivity (%)**	**95% CI**
≤ 100	23	69.6	[47.1–86.8]	69.6	[50.8– 88.4]	56.5	[34.5–76.8]
101–200	41	80.5	[65.1–91.2]	80.9	[68.9– 92.9]	63.4	[46.9–77.9]
201–500	28	89.3	[71.8–97.7]	89.3	[71.8–97.7]	82.1	[63.1–93.9]
501–1000	40	100	–	100	–	100	–
1001–5000	34	97.1	[85.1–99.9]	100	–	91.2	[76.3–98.1]
5001–50 000	58	100	–	100	–	98.3	[90.8–99.9]
> 50 000	11	100	–	100	–	100	–

PCR assays detected 11 false-negative results (11/202 or 5.4%) by microscopy and 7 false-negative results (7/202 or 4.2%) by both microscopy and the RDTs. Overall, the combined results of microscopy and PCR showed a sensitivity of 85.7% for Paracheck™-Pf, 85.4% for SD Bioline malaria Ag-Pf and 88.2% for SD Bioline Ag-Pf/Pan. The specificity was 86.0% for Paracheck™-Pf, 86.3% for SD Bioline malaria Ag-Pf and 80.4% for SD Bioline malaria Ag-Pf/pan.

The overall sensitivities, specificities, NPV and PPV for the three RDTs, evaluated against microscopy and microscopy and PCR are detailed in Table 2.

**Table 2:** Overall sensitivity, specificity and negative and positive predictive value of the three RDTs evaluated against microscopy, and microscopy and PCR

	**Reference**	**Sensitivity**	**Specificity**	**Positive predictive value**	**Negative predictive value**
		**[95% CI]**	**[95% CI]**	**[95% CI]**	**[95% CI]**
**Paracheck™-Pf**	Microscopy	85.9 [80.8-90.1]	86.6 [81.1-91.0]	88.2 [83.2-92.1]	84.1 [78.4-88.8]
	Microscopy and PCR	85.7 [80.8–89.8]	86.0 [80.1–90.6]	89.3 [84.7-92.9]	81.5 [75.4-86.7]
**SD Bioline AgPf**	Microscopy	92.3 [88.1-95.4]	82.2 [76.2-87.2]	85.7 [80.8-89.8]	90.2 [85.0-94.1]
	Microscopy and PCR	85.4 [80.5–90.7]	86.3 [80.5–9.7]	89.7 [85.1-93.2]	81.0 [74.8-86.3]
**SD Bioline AgPf/Pan**	Microscopy	92.3 [88.1-95.4]	81.2 [81.1-91.0]	85.0 [80.0-89.2]	90.1 [84.8-94.0]
	Microscopy and PCR	88.2 [83.2–92.0%]	80.4 [74.3–85.5]	83.1 [77.7-87.6]	86.1 [80.5-90.7]

## Discussion

The sensitivity and specificity of the tested RDTs found in this study appear to fall within the ranges quoted in other studies, which reported a sensitivity of Paracheck™-Pf of 82.9–100% and a specificity of 56.0–96.4% [34-42] and a sensitivity of the SD Bioline tests of 83.3–100% and a specificity of 86.8–98.9% [43-45]. WHO standards for RDT procurement for high-transmission areas include a recommendation that the panel detection scores against *P. falciparum* samples be at least 75% at 200 parasites/μl, although discrepancies between these scores and clinical sensitivity are limitations of these tests [46,47].

In this study, some blood samples that were negative by microscopic examination gave negative results in all three RDTs, while some gave positive results in all RDTs. Factors that affect the sensitivity and specificity of RDTs, such as low parasitaemia, are particular challenges for malaria diagnosis with these new devices. We found that a parasitaemia between 40 and 400 parasites/μl in blood samples with positive results by microscopy gave negative results in all three RDTs. This finding corroborates those of other studies [48-52], which reported false-negative results with RDTs at a parasitaemia < 500 parasites/μl. It has been suggested that lower levels of *HRP2* during a malaria episode with low parasitaemia might not be detected by RDTs [53] because malaria antigenaemia depends on the parasite biomass in the patient’s body during an acute episode [54].

Furthermore, deletions or mutations in the *HRP 2* gene may reduce the sensitivity of these RDTs [16-21]. It has also been speculated that the variation in *PfHRP2* sequences in samples from the Central African Republic might reduced the sensitivity of RDTs for detecting malaria at very low parasite density [29]. Other factors, such as the ‘prozone effect’ for Paracheck™-Pf at high parasite densities [55] and the presence of anti-*HRP 2* in humans [22], might explain why some tests give negative results despite significant parasitaemia.

Cases incorrectly identified as positive by the RDTs might be due to the persistence of *PfHRP2* from previous malaria infections [56], cross-reactivity with human autoantibodies [23,24] or other infectious diseases [25].

This study has some limitations. First, PCR was performed only on samples found negative by microscopy. It might be considered incorrect to use a different “gold standard” for slides found to be positive and those found to be negative by microscopy, by using PCR only for slides found negative by microscopy. However, the risk for false-positive microscopy results was considered low, as the slides were read independently by two experienced technicians. The fact that the microscopists counted only 200 negative leukocytes before declaring a slide as negative might have decreased the sensitivity of microscopic detection in this study. PCR is a useful gold standard because it is highly sensitive, can detect cases with low parasitaemia that are missed by other techniques and is easily reproducible [57]. Nevertheless, it is very expensive and time- and labour-consuming and is therefore used only to confirm the accuracy of microscopy in cases of sub-patent parasitaemia [58] and to evaluate the performance of the RDTs or microscopy in determining malaria prevalence [59].

## Conclusions

This study showed that the performance of SD Bioline malaria Ag-Pf and SD Bioline malaria Ag-Pf/pan was better than that of Paracheck™-Pf. SD Bioline malaria Ag-Pf/pan is the most useful test because it detects other species of *Plasmodium*, even if they are less prevalent in the Central African Republic. Moreover, introduction of these tests will indisputably help to identify malaria cases in this area, where microscopy is still of poor quality. Microscopy should nevertheless complement RDTs for determining parasite density, and therefore reinforcement of microscopy skills by ongoing training of technicians and quality assurance of both RDTs and microscopy should be considered priorities in national malaria programmes. The results of this study also indicate that RDTs should be evaluated in each new setting before they are deployed, in view of possible variations in performance in different populations.

## Competing interests

The authors declare that they have no competing interests.

## Authors’ contributions

DD and JMM conceived the study, with substantial contributions from AM and NM. DD, JI, JCG and GT participated in field data collection and laboratory analysis. AM, DD and SB interpreted the data and drafted the manuscript. All the authors have read and approved the final version.

## Pre-publication history

The pre-publication history for this paper can be accessed here:

http://www.biomedcentral.com/1471-2334/14/109/prepub
